# Sustainable Intensification of a Rice–Maize System through Conservation Agriculture to Enhance System Productivity in Southern India

**DOI:** 10.3390/plants11091229

**Published:** 2022-05-01

**Authors:** Mangal Deep Tuti, Mahender Kumar Rapolu, Banugu Sreedevi, Nirmala Bandumula, Surekha Kuchi, Sonth Bandeppa, Soumya Saha, Brajendra Parmar, Santosha Rathod, Gabrijel Ondrasek, Raman Meenakshi Sundaram

**Affiliations:** 1 ICAR-Indian Institute of Rice Research, Hyderabad 500030, India; kumarrm21364@gmail.com (M.K.R.); sreedevi.palakolanu@gmail.com (B.S.); surekhakuchi@gmail.com (S.K.); bgsonth@gmail.com (S.B.); birju1973@gmail.com (B.P.); santoshagriculture@gmail.com (S.R.); rms_28@rediffmail.com (R.M.S.); 2 ICAR-National Rice Research Institute, Cuttack 753006, India; saha.soumya5@gmail.com; 3 Faculty of Agriculture, University of Zagreb, 10000 Zagreb, Croatia; gondrasek@agr.hr

**Keywords:** conservation agriculture, rice–maize system, southern India, sustainable intensification

## Abstract

Integrated management of rice–maize systems is an emerging challenge in southern India due to improper rice residues and tillage management in maize crops. Conservation agriculture (CA) practices such as a reduced tillage and maintaining stubble mulch may hold the potential to increase yields, reduce crop establishment costs and increase farm incomes. A five-year trial was performed to study the effect of different CA and establishment methods in rice on system productivity, profitability, and soil carbon status in a rice–maize system. In the rainy season, the trial consisted of two main treatments: (i) normal manual transplanting and (ii) direct-wet seeding, and three sub-main treatments at different sowing dates with fifteen day intervals. In addition, in the winter season, two tillage treatments (conventional and minimum tillage) were imposed over the rainy season treatments. Both rice and maize were grown under irrigated conditions. The results showed that sowing times at 15 day intervals did not impact the yield significantly. Transplanted rice obtained a significantly higher grain yield during the first four years, but in the last year, the yield was similar in both of the establishment methods. In the winter season, conventional tilled maize recorded a higher cob yield than under the minimum tilled treatment, except for the last year, where both the tillage treatment effects were the same. System productivity of CA-based minimum tilled rice–maize was inferior during the first three years but was superior to the conventionally tilled method in the fourth and fifth year. Pooled analysis revealed that the conventionally tilled rice–maize system resulted in a similar system productivity as that of the CA during the study period. The cost–benefit analysis revealed that transplanted rice and conventionally tilled maize fetched higher net returns of INR 111,074 and INR 101,658/ha, respectively, over the direct-wet seeded rice and CA. In addition, the 15 July rice sown followed by the maize system led to an increase in irrigation water productivity by 15.7%, and the total water (irrigation + rainfall) productivity by 27.1% in the maize crop compared with the 30 July sown system. The CA-based rice–maize system resulted in a significantly higher very labile (0.194%) and labile (0.196%) carbon concentration at a 0–5 cm depth of soil compared to those under the conventional system. Thus, CA can be recommended for southern India and similar agro-ecological tropic and sub-tropic conditions. This system can be followed with appropriate location-specific modification in South-Asian countries, where crop yields and soil health are declining as a result of continuous cereal–cereal crop rotation.

## 1. Introduction

In India’s post-green revolution age, resource conservation has become more important due to the extensive deterioration of resource bases and the necessity to reduce ever-increasing production costs. Cereals are the world’s most important food and feed crops, with wheat, rice, and maize accounting for three-quarters of the total acreage [1]. Maize and rice with faster growth rates (3.0% and 2.3%, respectively) may be able to meet India’s growing food demand [2]; however, in intensively cultivated agricultural systems, there has been a significant overexploitation of natural resources, soil health degradation, and global warming impacts [3]. Rice is the staple food for over half of the global population [4] and in India, rice accounts for 40% of the total food grain production [5]. A rice–rice cropping system is usually practiced by farmers where sufficient irrigation is available or in favorable lowland rainfed areas [6]. In India, the rice–rice system occupies ~6.0 Mha and is becoming the most dominant cropping practice in South India; however, due to the continuous practice and adoption of the rice–rice system, several issues related to nutrient imbalance and/or deficiency, low nutrient-use efficiency, water scarcity/stress, energy and a labor crisis, high greenhouse gas emissions, and weed management [7] threaten the sustainability of this system. In wet fields under irrigated and deep-water conditions, direct seeding can be performed either through broadcasting or drilling seeds. Thus, wet direct seeded rice is becoming a sustainable alternative to transplanted rice cropping. It has been observed that differences in yield between wet direct seeded rice and transplanted rice increase with a reduced water input.

Conservation agriculture (CA) is seen as a feasible approach for crop intensification that is both sustainable and profitable [8,9,10]. To maximize the benefits of CA, location-specific appropriate crop rotations and system-based CA strategies must be devised [8,9]. This necessitates sufficient on-station and on-farm research in order to establish location-specific CA practices [11,12]. Some CA-based components, such as zero tillage (ZT), crop residue retention, and crop diversification, have been investigated in South India as an alternative to conventional tillage [13]. One of the main goals of CA techniques is to increase the concentration of soil organic carbon (SOC) [14]. In fact, the amount and quality of crop residues added to the soil have a considerable impact on SOC sequestration and stabilization in CA [15,16]. More residue additions and/or lower tillage intensity in a cropping system over time sustains or even raises SOC levels [17]. Maize is grown throughout the dry winter months and has a deep tap root structure, therefore it requires less water. Maize uses less water for irrigation than rice, resulting in water saving and increased water efficiency [18]. In both irrigated and rain-fed agricultural settings, maize can be a cost-effective alternative to winter rice. The Government of India (GOI) recently announced a program to promote maize by diversifying rice–rice areas, which are extensively spread in irrigated agroecosystems in eastern, central, peninsular, and southern India. In fact, in irrigated settings, CA with ZT/minimum tillage (MT) and residue retention is preferable for growing maize following rice [19]. The land remains unoccupied from October to the middle of November after the harvest of the rainy rice crop in September–October. As a result, alternative establishment strategies can be used to change the planting timing of rainy season rice in June–July. With various crop cycles such as the rice–maize systems, there is ample possibility to adopt CA in southern India. To test the viability of a rice–maize system with CA practice, we used three sowing periods, two establishment methods, and two tillage treatments in this study; however, data on the effects of CA on productivity, profitability, and resource-use efficiency in rice–maize rotation, all of which are crucial sustainability indicators for such a system, are scarce. We hypothesized that a CA-based practice consisting of wet direct seeded rice followed by MT in winter season maize would result in higher crop productivity, profitability, water-use efficiency, and soil carbon content in rice–maize rotations than a system consisting of transplanted puddled rice followed by conventional tilled maize. The study’s goals were to (i) evaluate the medium-term (5-year) impacts of CA on test crops and system productivity, sustainability, and economics; and (ii) quantify water-use efficiency and soil carbon stocks in the rice–maize system in southern India.

## 2. Materials and Methods

### 2.1. Experimental Site

The experiments were carried out at the ICAR-Indian Institute of Rice Research, Hyderabad, Telangana (11°00′ N; 77°00′ E, 427 m above sea) during the period 2016 to 2021 continuously with a sub-tropical and semi-arid climate. During the period of the study, May was the hottest (38.3–45 °C), and December the coolest (9–11 °C) month. In the study area, the average annual precipitation was 710 mm, with roughly 80% falling during the southwest monsoon season (July–September) and the rest falling during the ‘Western Disturbances’ (December–February). Experimental soils were classified as vertisols. The initial soil properties of the experimental plot are given in Table 1.

### 2.2. Treatments and Crop Management

The experimental field was uniformly leveled by a tractor-drawn land leveler in September 2015. A green manure crop (*Sesbania aculeata*) was grown in March 2016 to make the experimental soil more fertile, and during the 2016 wet season, the experiment was initiated with a rice crop. From 2016–2017 through 2020–2021, the rice–winter maize sequence was studied for five years, involving seven conservation and conventional treatments. The RNR 15,048 (Telangana Sona), a fine rice variety with 120–125 days vegetation duration, having a tolerance to bacterial leaf blight and suitable for late sowing (July) was selected as a test crop in the rainy season, whereas DHM 117, a promising medium to late maturing single cross maize hybrid was selected as a test taken as a winter crop. The applied treatments and an experimental layout were fixed from the beginning of the CA experiment in 2016–2021, which continued up to 2020–2021. Each plot was 11 m × 6.0 m, and the experiment was laid out in a split-plot design in the rainy season. Further two tillage treatments (conventional tillage and minimum tillage) were imposed in the winter season by dividing each plot of the rainy season into two equal halves. A maize crop was sown on 16 November of every year. Therefore, the rice–maize system as a whole was laid out in split–split design which included both the rainy and winter seasons with four replications. Sowing was completed in 3 phases as per the treatments. For the wet direct seeded rice (W-DSR) a 20 kg/ha seed rate was used. Twenty-five days old rice seedlings were transplanted manually in transplanted puddled rice (TPR) with two seedlings per hill at a 20 cm × 10 cm row geometry, having a plant density of 50 hills/m^2^. In the rainy season the main plots were ploughed thoroughly and a 2 cm thin film of water was maintained for transplanting; however, for the W-DSR, the plots were puddled and kept saturated before manual broadcasting. The maize seeds were sown at the rate of 20 kg/ha. The spacing between the maize crop was 0.60 m × 0.25 m.

The rice received a recommended dose of 120 kg N, 26.2 kg P, and 33 kg K/ha, respectively, from urea (46% N), single super phosphate (46% P_2_O_5_), and muriate of potash (60% K_2_O). At the active tillering and flowering stages, 50% nitrogen was applied as a basal dose, and the remaining 50% was applied in two split doses of 30 kg/ha each. The maize crop was treated with 150 kg of nitrogen, 26.2 kg of phosphorus, and 33 kg of potassium per hectare. Before sowing/transplanting the rice, the complete amount of P and K and half of the total N were applied. At the tillering and panicle initiation stages of the rice, the remaining N was applied in two equal splits. The complete amount of P and the K and half of the total N were applied before sowing of the maize crop. At the knee-high and tasseling stages of the maize, the remaining N was applied in two equal halves.

Glyphosate 1.0 kg a.i./ha (41% SL) was applied to the MT plots before seeding the maize crop to control the existing grassy, broad-leaved, and sedge weeds. Pendimethalin 1.0 kg a.i./ha at two days after sowing (DAS) and bispyribac-Na (10% SC) 25 g a.i./ha at 23 days after sowing were used to control weeds in the W-DSR (Das, 2008). Pendimethalin 1.0 kg a.i./ha was used to control weeds in the transplanted rice and maize crops at two days after transplanting (DAT) or sowing (the volume rate of clean water was 400 L/ha).

### 2.3. Rice and Maize Grain Yields and Weed Population

For grain yields, a net area of 5 m × 2 m, consisting of 10 rows of rice and 5 rows of maize up to a length of 5 m, was harvested from the middle locations of each plot at maturity. W-DSR matured rice was harvested in the second week of October (12–15 October) for the 1 July sowing, whereas TPR was harvested after seven days (20–22 October) in each year. Similarly, the harvested dates were 15 days later for the 15 July sowings and another 15 days later for the 30 July sowings, according to the sowing procedures. The same plot was prepared for the tillage treatment and the winter maize was sown as soon as the rainy season rice crop was harvested. As a result, there were three separate sowings in all. The rice crop was manually harvested from the no residue plots at a height of 5 cm above the ground. Rice and maize were harvested at 30 cm height in the residue retention plots. After the maize crop was harvested, glyphosate was applied at a rate of 1.0 kg/ha to the crop residue, which was then dried and decomposed. The rice and maize cob grain yields were measured at 12% moisture content. Rice straw yields and maize stover yields were calculated after oven drying the samples to a constant weight at 70 °C and expressed on a dry weight basis. Equation (1) was used to convert the maize cob yields to a rice equivalent yield (REY). Minimum support prices (MSP) for the rice and maize were accounted as per the Government of India’s declaration [20]:

REY of maize = [(Maize yield × price of maize)/(price of rice)](1)

Throughout the growing cycle of the rice and maize crops, the emergence of weeds in all plots of all treatments was documented, and the weed species were identified. Two quadrats (0.5 m × 0.5 m) in each plot were randomly chosen to encompass one central row of maize to determine weed density at 45 DAS. The number of weed species found in these quadrats was tallied and classified as monocot grassy, monocot sedge, and dicot/broad-leaved weeds, and the results were expressed as a number/m^2^. The total weed density was calculated by adding these categories/classes of weeds together.

### 2.4. Sustainable Yield Index

The Equation (2) was used to calculate the sustainable yield index of rice, maize, and the rice–maize system over a five-year period, as per [21]:

(Yt − σ)/Ymax = Sustainable Yield Index (2)

where Yt is the mean grain yield in a certain treatment or system, and σ is the standard deviation of a treatment with the highest yield in the treatments and over time (Ymax).

### 2.5. Economics

All inputs/operations required for employing/imposing a particular treatment in the rice and maize crops, such as seed, crop residue, tillage (MT, CT, puddling/direct-sowing), nursery-raising, transplanting, fertilization, irrigation, plant protection measures, harvesting, and threshing were listed, and their current market prices were added up to estimate the cost of cultivation of that treatment. For each crop, the cost of hiring tractor-driven machinery and the wage of human laborers (based on eight hours of work per day) were also factored in to determine the cost of cultivation. The government of India’s minimum support price (MSP) for rice and maize was utilized to calculate the economics [22] and was used to calculate the gross return. As a result, the net return was determined using Equation (3):

Net returns = Gross returns − Cost of cultivation (3)

### 2.6. Water Productivity

The depth of applied irrigation water was determined using a digital water meter and the advancement of wetted area in the irrigation channel. We first created a rating curve based on the depth of water flow and discharge in the main channel, and then devised an exponential equation [23] to aid in the calculation of flow depth per irrigation treatment. Simultaneously, the periodical soil moisture content before irrigation was determined using the time domain reflectometer [18], which was also used to determine the frequency and quantity of water. Irrigation water may have been applied to the crops once the available soil moisture at the root zone had been depleted by 50%. The depth of irrigation water was decided as per [24].

The depths of the root zone across the growth phases were surpassed by [25]. The rice crop was watered on a weekly basis. In addition to the rainfall obtained during the crop growth period, the maize crop received ten irrigation treatments. Effective rainfall was calculated using the total rainfall data from a rain gauge for each crop season [26]. The total amount of water applied to each treatment was calculated by adding the irrigation water applied to the crops and the effective rainfall. Following [25], the water productivity (i.e., footprint) was estimated by dividing the grain yield by the total water used in each treatment.

### 2.7. Carbon Fractions in Soil

Soil samples were taken with a core sampler from the 0–5 and 5–15 cm soil layers after the 5th crop cycle (i.e., after the maize crop was harvested) in the last week of April 2021. Six soil cores were taken from each depth, from the center rows of each plot to avoid the border effect, and thoroughly mixed to create a composite sample for carbon content assessment. Before analysis, the samples were air-dried for 72 h and sieved at 2.0 mm. The dry combustion method [26] was used to test 48 samples (12 treatments × 4 replications) using a TOC analyzer (Elementar Vario Select, Hanau, Germany), as detailed in [27]. Briefly, the lability-graded carbon fractions were determined using the modified Walkley and Black method [28,29] with 5, 10, and 20 mL of concentrated sulphuric acid (H_2_SO_4_), yielding three acid aqueous solution ratios of 0.5:1, 1:1, and 2:1, respectively. Following that, four distinct carbon fractions were obtained: very labile carbon (i.e., the part of organic carbon oxidized in 5 mL H_2_SO_4_), labile carbon (organic carbon oxidized in 10 mL H_2_SO_4_—organic carbon oxidized in 5 mL H_2_SO_4_); less labile carbon (organic carbon oxidized in 20 mL H_2_SO_4_—organic carbon oxidized in 10 mL H_2_SO_4_); and non-labile carbon (total organic carbon—organic carbon oxidized in 20 mL H_2_SO_4_).

### 2.8. Statistical Analysis

Using PROC GLM in the statistical software package SAS 9.3, data on rice, maize, and weed were evaluated using the analysis of variance (ANOVA) approach for split plot (rainy season) and split–split (winter season) designs [30] (SAS Institute, Cary, NC, USA). Weed population data were not converted because there was no difference in the test of significance between the observed and transformed data, and ANOVA was performed using the original/observed data [31]. The pooled ANOVA was also used to determine the effects of different treatments and their interactions on rice and maize grain yields, as well as system productivity. At a 5% level of significance (*p* = 0.05), Tukey’s honest significant difference test was performed as a post hoc mean separation test.

## 3. Results

### 3.1. Rice, Maize, System Productivity, and Economics

#### 3.1.1. Rice Grain Yield

There was no significant difference among the different dates of sowings across the years (Table 2). Between the establishment methods, the yield obtained in transplanted rice was significantly higher than in the W-DSR, in all years except 2020. Further, the grain yield of transplanted rice increased until the third year, then declined in the fourth and fifth years (Table 2), whereas the grain yield of W-DSR remained stable throughout the years. With the exception of 2017, the highest rice yield was reported in the 15 July sowing in 2018 (6.31 t/ha) out of all five years of the study. The transplanted rice yielded much more in the third year (6.31 t/ha) than the other two establishment methods. According to pooled analysis, the transplanted rice produced 11.51% more grain yield than the W-DSR rice (Table 2).

#### 3.1.2. Maize Grain Yield

In general, the maize cob yield gradually decreased as the years progressed, with the highest yield recorded in 2016–2017, and the lowest in 2020–2021 irrespective of the rainy season sowing time, establishment methods and winter imposed tillage treatments (Table 3). The rice crop sown in 15 July had a significant effect on the winter maize yield. During the initial three years (2016–2017 to 2018–2019), the residual effect of the rainy season transplanting method of crop establishment had a significant effect on cob yield of the winter maize compared to that under the W-DSR (Table 3); however, the latter two years of the rotation maize yield was found to be similar under both crop establishment methods. The winter season imposed by the conventional tilled treatment resulted in a significantly higher cob yield in the initial four years (Table 3). In addition, pooled analysis confirmed similar results; however, in the last year (2020–2021) the minimum tilled plots produced a comparable yield with the conventional tilled plots (Table 3). The transplanted rice and conventional tilled maize resulted in 10.6% and 17.3% higher maize cob yield than that of the W-DSR and minimum tilled plots, respectively (Table 3).

#### 3.1.3. Rice–Maize System Productivity

The rice crop sown on 15 July significantly resulted in the highest system productivity in 2016–2017 (13.27 t/ha) and 2017–2018 (12.76 t/ha) (Table 4); however, in the latter years (2018–2019, 2019–2020 and 2020–2021) the sowing time did not affect the system productivity. The transplanted rice-based system productivity was superior over the wet direct seeded system during all the five years of experimentation (Table 4). The highest system productivity of transplanted rice was recorded in 2018–2019 (12.63 t/ha). The conventional tilled maize-based system was superior over the minimum tilled maize in the initial three years. The highest system productivity of the conventional tilled maize system was recorded in 2018–2019 (12.54 t/ha). In the latter two years there was no significant difference between the conventional tilled maize and minimum tilled maize systems. Pooled analysis also reflected the same results as that of last two years (Table 4).

#### 3.1.4. Economics of the Rice–Maize System

In respect to the establishment method, the five-year mean cost of cultivation under the wet direct seeded system was less than that of the transplanted rice by INR 3000/ha (Table 5). Maize cultivation in the minimum tillage plots cost INR 3000/ha less than the conventional tilled maize agriculture (Table 5). As a result, the overall cost of cultivation in the transplanted rice–maize system was greater than the wet direct seeded rice–maize system (by INR 4000/ha). The transplanted rice, on the other hand, yielded a greater additional net return of INR 6468/ha than the wet direct seeded rice. Though the cost of cultivation of the maize crop was the same under the different establishment methods of previous rainy seasons, the net returns of the maize crop was higher (by INR 9713/ha) in the transplanted rice-based maize crop than in the wet direct seeded maize crop. System economics analysis reflected the higher net returns of the transplanting-based system (INR 111,074/ha) over the wet direct seeded system (INR 99,093/ha). Thus, there was a net profit of INR 11,978/ha due to the transplanting method than the wet direct seeded method. Similarly, the cost of cultivation of the minimum tilled-based rice–maize system was lesser (by INR 3000/ha) than that of the conventionally tilled-based rice–maize system. Contrary to this, the conventionally tilled maize resulted in higher net returns of INR 12,314/ha over the minimum tilled maize cultivation. The net return of the conventional tilled maize system was higher (INR 114,352/ha) than the minimum tilled maize system (INR 101,658/ha) (Table 5).

### 3.2. Sustainable Yield Index of Rice, Maize and Rice–Maize System

Over a five-year period, the sustainable yield index of the rice, maize and rice–maize systems changed significantly between the conventional and conservation agriculture approaches (Table 6). The transplanted rice had the highest sustained yield index during the rainy season. Similarly, when compared to the minimum tilled winter maize, the traditional tilled winter maize yielded a higher sustainable yield index. When compared to all other systems, the transplanted rice–conventional tilled maize systems had significantly higher sustainable yield indexes of rice. The sustainable yield index is highly correlated with the water productivity of any cropping system, and here in this study it was clearly reflected that the water productivity of the maize crop in the rainy season transplanted rice crop was higher than the wet direct seeded rice. Further, due to residue retention in the minimum tilled plots, the total water productivity in the maize was increased compared to that of the conventional residue removal treatment (Table 7).

### 3.3. Weed Population, Rice and Maize Water Productivity

*Echinochloa crusgalli* (L.) Beauv., *Echinochloa colona* (L.) Link., *Cyperus esculentus* L., *Cyperus rotundus* L., and *Parthenium hysterophorus* were among the weed flora that appeared in the rice–maize system over time. Three weeds, *E. colona*, *C. rotundus*, and *C. esculentus*, were found to be absent throughout the five-year research under the traditionally tilled transplanting system. *D. Aegyptium*, *E. colona* and *C. rotundus*, on the other hand, were present in every year under the wet direct seeded minimum tilled system. In the transplanted rice, *Echinochloa crusgalli* was present for all five years, but was absent for the first three years and only appeared in the fourth year onward in the wet direct seeded rice. Similarly, *Melilotus indica* (L.), *Anagallis arvensis* L., *Coronopus didymus* (L.) Smith, *Chenopodium album* L., and *Rumex dentatus* L. infested the conventionally tilled and minimally tilled maize. During all five years, *A. Arvensis*, *M. indica*, *Rumex dentatus* and *C. album*, occurred simultaneously in both the conventionally tilled and minimally tilled plots. Regardless of treatment, the total weed population rose as the years continued (Table 8). Due to the diverse sowing dates, no apparent trend in the weed flora was identified. Furthermore, in a pooled analysis, the overall weed population was determined to be non-significant. In all five years of the winter maize cropping, the wet direct seeded rice-based plots had a larger overall weed population than the transplanted rice-based plots. Pooled analysis also reflected the same result. The total weed populations were lower in the conventional tilled maize plots than the minimum tilled plots in all five years. The highest total weed population was 146 n/m^2^ in the minimum tilled plots in the year 2020–2021 (Table 8).

Generally, the maize crop showed higher irrigation water productivities than the rice crop (Table 7). Further, among the different dates of sowing, the maize crop recorded the highest irrigation water productivity (7.21) for 15 July sowing date (Table 7). The same result was also reflected in terms of total water productivity (5.34) under the same sowing time. The different establishment methods did not affect either the irrigation water productivity or total water productivity in either crops; however, the minimum tilled maize crop showed a significantly higher total water productivity (5.33) over the conventionally tilled maize plots (4.89). On average, the residue retention in the minimum tilled plots was found to be superior, which increased the total water productivity by 9% in the maize over the conventional residue removal treatments (Table 7).

### 3.4. Soil Carbon Content

The different dates of sowing and establishment methods did not impact the soil carbon pools (Table 9). The minimum tilled maize system had a significant impact on the very labile and labile carbon pools, but not on the less labile and non-labile carbon in the 0–5 cm soil layer (Table 9). The minimum tilled maize resulted in significantly higher very labile (∼33.5%)and labile (∼33.2%) carbon concentrations at a 0−5 cm depth of soil compared to the conventionally tilled maize system; however, in the deeper (5–15 cm) soil layer, none of the pools of carbon (very labile, labile, less labile and non-labile) differed significantly between the treatments.

The maize yield in both the conventionally tilled and minimally tilled plots declined significantly after the first year, indicating that the soil fertility had diminished. Under the minimum tilled plots, the mean soil profile (0–30 cm) moistures were 12.1% and 12.9% in 0–5 and 5–15 cm soil, respectively, after the maize sowing (Table 10).

Under the minimum tilled plots, the mean soil profile (0–30 cm) moistures were 12.1% and 12.9% in 0–5 and 5–15 cm soil, respectively, after the maize sowing (Table 10).

## 4. Discussion

### 4.1. System Productivity, Sustainability, and Profitability

In South India’s agroecosystems, a transplanted rice–rice system has been advocated to boost the input-use efficiency and profitability. Rice–maize rotation based on conservation agriculture can replace the rice–rice system in some parts of southern India and is better suited to irrigated environments. Our prediction that CA-based rice–maize rotations might eventually replace transplanted rice–maize in southern India has been validated (e.g., Table 4). The wet direct seeded rice establishment method resulted in a slightly lower rice yield (by ∼0.5 t/ha) than transplanted rice in all the initial four years but had a comparable yield in the fifth year (Table 2). Similarly, the fifth year minimum tilled maize crop showed encouraging results by producing a similar cob yield with conventionally tilled maize, though the initial four years results were different (Table 3). A higher maize yield obtained in a sustainable way was the determining factor that increased the rice–maize system yields in the later stage under the conservation agriculture-based wet direct seeded rice, minimum tilled rice–maize system over the conventional system, though the five year mean net returns showed a contrasting result. The transplanting rice system also provided a higher yield and net income. In the long run, a W-DSR and minimum tilled rice–maize system will be a viable option to achieve higher net returns as the fifth year yield results reflected the same. Due to decreased costs in tillage/equipment, labor, fuel and irrigation, the conservation agriculture technique had a lower cost of cultivation per hectare than the transplanted conventional rice–maize system by INR 7000/ha.

Allelopathic effects of rice and maize residues on subsequent crops are possible [32,33]. The declining maize yield in both the conventionally tilled and minimally tilled plots indicate that the soil fertility had diminished. Such results could indicate that the maize crop was absorbing more nutrients or that the appropriate fertilizer dose needed to be raised.

In the maize plots, frequent tillage after a winter conventional tillage resulted in an increased evaporation loss at 0–15 cm depth of soil compared to the minimum tilled plots (Table 9). Because of having no or minimal soil disturbance and residue retention (Table 9), the minimum tilled plots had an increased surface (0–5 cm) soil moisture, which aided the maize seedling germination and stand establishment; however, the rice yield was comparable to that of a wet direct seeded rice-based system when transplanted procedures were used.

Due to puddling, continued submergence, and subsequent herbicide application, the transplanted rice exhibited lesser weed interference, resulting in a better rice yield than the W-DSR [34]. Repeated weed flushes in the W-DSR, on the other hand, avoided efficient weed control by herbicides [35], giving the weeds more competition for sunshine, nutrients, and space with the rice, resulting in poorer yields (Table 2). Puddling boosted the water holding capacity in transplanted rice, and there was 2.0–2.5 cm of standing water throughout the growing period, entirely alleviating water stress and resulting in a better rice yield in the transplanted rice than the wet direct seeded rice. In the final year of the trial, however, the CA-based W-DSR produced equivalent rice yields to the transplanted system and the typical tilled plots produced a more sustainable maize yield.

Similar to the W-DSR system, this resulted in higher sustainable rice–maize system productivity. In the fourth and fifth years of the study, the crop establishment and tillage methods, as well as weed and water management, dramatically enhanced the rice and maize yields in the CA-based minimum tilled systems (Table 3). In general, intensive conventional tillage without biomass residues and puddling causes a depletion of soil carbon and nutrients [36,37], underpinning water repellency in the soil [38]. After planting seedlings, for example, soil becomes hard and sticky, forming large clods and forcing repeated tillage (5–6 ploughs in average) to loosen/crumble the soil and obtain a fine top soil structure [39]. As a result, maize or other crops cultivated in succession are delayed in sowing. Furthermore, surface soil loses moisture quickly under tillage circumstances (vs. little tillage), resulting in poor germination, uneven field stands, and reduced crop growth/yields. Reduced tillage circumstances also seem to improve soil biodiversity [35]. Crop residues improve the balance between macro- and micro-porosity [40], allowing maize roots to develop and biomass to accumulate. Higher water productivity is also achieved as a result of irrigation water saving, i.e., higher water usage efficiency [18] and agricultural productivity [41]. In the CA-based minimal tilled practices, the total amount of water applied is lower than in a transplanted rice-based system. Crop remains on the surface may slow soil evaporation and hence save more soil moisture than having no residue [18]. This research also shows that minimal tillage combined with a continuous soil layer of residue can impact weed density and dynamics over time [42]. For example, *E. colona*’s ecological preference for aerobic conditions results in an increased infestation in W-DSR-based systems. *C. rotundus*, on the other hand, is a perennial weed and because underground tubers germinate more readily under aerobic circumstances, *C. rotundus* is more common in undisturbed soils. Anaerobic conditions induced by puddling and persistent standing water in the transplanting system, on the other hand, result in tuber dormancy and decreased infestation [43]. The W-DSR was grown throughout the rainy season, when the surface soil remained moist, resulting in 3–4 additional flushes of weed germination and a reduction in the effect of residue retention.

### 4.2. Carbon Content of Soil

Because this study was conducted over a short period of time (five years), and in sub-tropical conditions with high temperatures, it was unable to demonstrate a substantial impact of CA and CT practices on all four carbon pools across the soil depth (Table 10). Only the top soil (0–5 and 0–15 cm) layers had substantial differences in carbon stock. In the CA-based minimum tilled maize system, higher biomass carbon inputs through crop residues for five years led to significantly higher very labile and labile carbon pools than in the conventionally tilled system. Elsewhere, [41] found a similar increase in labile carbon in CA practice compared to a traditional tilled system, for example, the labile carbon pool has the fastest turnover rates, and its oxidation is what drives the carbon dioxide transport from soil to the atmosphere. Bigger top soil microbial populations in residue-amended plots may quickly metabolize carbon and nitrogen provided by the residue, resulting in a higher labile carbon pool without influencing the non-labile pool [44]. The non-labile and less labile carbon pools were not significantly different between the conventional and CA-based minimum tillage systems in this study. The rice residue was silica-rich and resisted decomposition, but the maize residue was hollow, light-weight, and allelopathic. CA, on the other hand, could mitigate the negative impacts of continual tillage by speeding up soil aggregation and carbon absorption [12].

## 5. Conclusions

In the fourth and fifth years of rotation, the CA-based minimum tilled maize system produced a lower system yield; however, this was similar to that of the conventionally tilled maize system. Furthermore, the CA-based wet direct seeded rice followed by the minimal tilled maize system had higher net returns than the transplanted rice followed by the traditional tilled corn system. In terms of system productivity, the CA-based minimum tilled maize system was comparable to the standard rice–maize system, but with lower cultivation expenses. In the rice–maize cycle, the CA (vs. conventional) system had higher water productivity, which resulted in higher highly labile and labile carbon concentrations in the topsoil. As a result, in conventional rice–rice and rice–maize rotations in southern India and similar (sub) tropical agroecosystems, the CA-based rice–minimum tilled maize system may be recommended for higher crop productivity, resource-use efficiency, and soil carbon in southern India and similar (sub) tropical agroecosystems. In rice–maize and other cropping systems, residue characterization and putative allelopathic impacts on crop growth must be investigated further.

## Figures and Tables

**Table 1:** Initial soil parameters of the experimental plot.

Parameter	Value
pH	8.23
EC (dS/m)	0.28
Organic carbon (%)	0.515
Available N (kg/ha)	206.3
Available P (kg/ha)	12.7
Available K (kg/ha)	210.6
Soil texture	clay
Exchangeable K (meq/100 g)	0.305
Exchangeable Na (meq/100 g)	0.240
Exchangeable Ca (meq/100 g)	5.355
Exchangeable Mg (meq/100 g)	1.773
CaCO_3_ equivalent%	1.048
Carbonate Carbon%	0.118
CEC (meq/100 g)	7.904
Chlorides (meq/100 g)	3.18
Sulphur (mg/kg)	9.93
Boron (mg/kg)	0.513
Zinc (mg/kg)	0.655
Copper (mg/kg)	0.242
Iron (mg/kg)	5.32
Manganese (mg/kg)	6.33

**Table 2:** Rice grain yield under conservation agriculture over the years.

Treatment	Rice Grain Yield (t/ha)					
	2016	2017	2018	2019	2020	Pooled
Sowing time						
1 July	5.43	5.48	5.80	5.60	5.52	5.57
15 July	5.80	5.62	6.31	5.88	5.68	5.86
30 July	5.60	5.70	6.10	5.80	5.60	5.76
LSD (*p* = 0.05)	NS	NS	NS	NS	NS	NS
Establishment method						
Transplanting	6.02	6.21	6.31	6.06	5.91	6.10
Wet direct seeded	5.40	5.39	5.53	5.46	5.59	5.47
LSD (*p* = 0.05)	0.52	0.54	0.57	0.51	NS	0.54

**Table 3:** Maize cob yield obtained under applied treatments during the 5-year period.

Treatment	Maize Cob Yield (t/ha)					
	2016–2017	2017–2018	2018–2019	2019–2020	2020–2021	Pooled
Sowing time						
1 July	6.82	6.73	6.44	6.01	5.78	6.36
15 July	6.88	6.65	6.40	5.88	5.64	6.29
30 July	6.54	6.23	6.02	5.73	5.51	6.01
LSD (*p* = 0.05)	NS	NS	NS	NS	NS	NS
Establishment method						
Transplanting	6.84	6.72	6.51	6.02	5.76	6.37
Wet direct seeded	6.15	6.01	5.81	5.52	5.31	5.76
LSD (*p* = 0.05)	0.66	0.61	0.63	NS	NS	0.60
Tillage (Winter season)						
Conventional	7.21	6.91	6.74	6.27	5.70	6.57
Minimum	6.06	5.81	5.47	5.37	5.28	5.60
LSD (*p* = 0.05)	0.67	0.62	0.64	0.68	NS	0.60

**Table 4:** Rice–maize productivity (t/ha) obtained under applied treatments during the 5-year period.

Treatment	Rice–Maize Productivity (t/ha)					
	2016–2017	2017–2018	2018–2019	2019–2020	2020–2021	Pooled
Sowing time						
1 July	11.76	11.67	12.06	11.43	11.24	11.63
15 July	13.27	12.76	12.62	11.58	11.23	12.29
30 July	11.67	11.43	11.95	11.36	11.06	11.49
LSD (*p* = 0.05)	1.13	1.11	NS	NS	NS	NS
Establishment Method						
Transplanting	12.37	12.39	12.63	11.90	11.61	12.18
Wet direct seeded	11.11	10.92	11.71	11.81	10.85	10.97
LSD (*p* = 0.05)	1.16	1.13	0.98	1.03	1.06	1.10
Tillage (Winter Season)						
Conventional	12.36	12.05	12.54	11.84	11.32	12.02
Minimum	11.29	11.04	11.31	10.97	10.90	11.10
LSD (*p* = 0.05)	1.01	1.00	1.10	NS	NS	NS

**Table 5:** Economics of rice, maize and rice–maize system during the 5-year period.

Treatment	Cost of Cultivation (INR/ha)			Net Returns (INR/ha)		
	Rice	Maize	System	Rice	Maize	System
Sowing Time						
1 July	48,560	38,650	87,210	45,643	63,586	109,229
15 July	48,560	38,650	87,210	50,564	62,411	112,974
30 July	48,560	38,650	87,210	48,900	57,984	106,884
Establishment Method						
Transplanting	54,650	38,650	93,300	48,462	63,812	112,274
Wet direct seeded	50,650	38,650	89,300	41,994	54,099	96,093
Tillage (Winter Season)						
Conventional	48,560	39,850	88,410	48,749	65,603	114,352
Minimum	48,560	36,850	85,410	48,369	53,289	101,658

**Table 6:** Sustainable yield index of rice, maize, and rice–maize system.

Treatment	Rice	Maize	Rice–Maize System
1 July	0.93	0.87	0.94
15 July	0.89	0.84	0.86
30 July	0.91	0.86	0.93
Transplanting	0.94	0.86	0.93
Wet direct seeded	0.92	0.81	0.93
Conventional tillage		0.85	0.94
Minimum tillage		0.81	0.91

**Table 7:** Irrigation water productivity and total water productivity of rice and maize crops during the 5-year period.

Treatment	Irrigation Water Productivity (kg Grain/m^3^)		Total Water Productivity (kg Grain m^3^)	
	Rice	Maize	Rice	Maize
Sowing Time				
1 July	3.86	7.03	2.02	5.13
15 July	4.22	7.21	2.31	5.34
30 July	4.06	6.23	1.98	4.20
LSD (*p* = 0.05)	NS	0.66	NS	0.51
Establishment Method				
Transplanting	4.03	6.88	2.12	4.92
Wet direct seeded	4.05	6.76	2.08	4.86
LSD (*p* = 0.05)	NS	NS	NS	NS
Tillage (Winter Season)				
Conventional	4.04	6.62	2.1	4.89
Minimum	4.04	7.02	2.1	5.33
LSD (*p* = 0.05)	NS	NS	NS	0.42

**Table 8:** Total weeds population at 45 days after sowing in maize during the 5-year period.

Treatment	Total Weed Population (Number/m^2^)					
	2016–2017	2017–2018	2018–2019	2019–2020	2020–2021	Pooled
Sowing Time						
1 July	61	78	87	112	132	94
15 July	58	71	82	124	128	93
30 July	54	74	78	132	124	92
LSD (*p* = 0.05)	5.2	NS	8.2	12.3	NS	NS
Establishment Method						
Transplanting	42	62	80	112	120	83
Wet direct seeded	72	88	94	132	136	104
LSD (*p* = 0.05)	8.6	8.8	10.2	13.4	13.6	11
Tillage (Winter Season)						
Conventional	51	64	78	102	110	81
Minimum	63	84	86	142	146	104
LSD (*p* = 0.05)	6.4	8.8	8.6	18.4	21.6	13

**Table 9:** Soil organic carbon (%) pools (0–5 cm and 5–15 cm soil layers) in rice–maize cropping system labile carbon during the 5-year period.

Treatment	0–5 cm				5–15 cm			
	Very Labile	Labile	Less Labile	Non-Labile	Very Labile	Labile	Less Labile	Non-Labile
Sowing Time								
1 July	0.182	0.183	0.089	0.181	0.114	0.042	0.112	0.328
15 July	0.187	0.189	0.078	0.164	0.116	0.046	0.113	0.320
30 July	0.193	0.192	0.082	0.168	0.113	0.048	0.110	0.346
LSD (*p* = 0.05)	NS	NS	NS	NS	NS	NS	NS	NS
Establishment Method								
Transplanting	0.185	0.186	0.080	0.170	0.112	0.042	0.112	0.361
Wet direct seeded	0.189	0.190	0.086	0.172	0.116	0.048	0.110	0.301
LSD (*p* = 0.05)	NS	NS	NS	NS	NS	NS	NS	NS
Tillage (Winter Season)								
Conventional	0.180	0.180	0.08	0.168	0.118	0.052	0.111	0.351
Minimum	0.194	0.196	0.086	0.174	0.121	0.051	0.111	0.311
LSD (*p* = 0.05)	0.01	0.01	NS	NS	NS	NS	NS	NS

**Table 10:** Soil moisture content (%) at 0–15 cm depth of soil at 5 days after sowing of maize during the 5-year period.

Treatment	Soil Moisture Content (%)		
	0–5 cm	5–15 cm	15–30 cm
Sowing time			
1 July	11.2	12.6	13.6
15 July	11.6	12.2	13.4
30 July	10.2	12.0	13.0
LSD (*p* = 0.05)	NS	NS	NS
Establishment method			
Transplanting	10.8	12.2	13.1
Wet direct seeded	11.2	12.4	13.3
LSD (*p* = 0.05)	NS	NS	NS
Tillage (Winter season)			
Conventional	10.0	11.5	13.0
Minimum	12.1	12.9	13.6
LSD (*p* = 0.05)	1.0	1.0	NS

## Data Availability

Data are contained within the article.
